# The complete chloroplast genome of *Epimedium sagittatum* (Sieb. Et Zucc.) Maxim. (Berberidaceae), a traditional Chinese herb

**DOI:** 10.1080/23802359.2019.1640087

**Published:** 2019-07-16

**Authors:** Xiang Liu, Qianru Yang, Cheng Zhang, Guoan Shen, Baolin Guo

**Affiliations:** a Chongqing Academy of Chinese Materia Medica, Chongqing, China;; b Institute of Medicinal Plant Development, Chinese Academy of Medical Science, Peking Union Medical College, Beijing, China

**Keywords:** Chloroplast genome, *Epimedium sagittatum*, Berberidaceae

## Abstract

*Epimedium sagittatum* is an important traditional medicinal plant in China. In this study, we assembled the complete chloroplast (cp) genome of *E. sagittatum*. The whole cp genome of *E. sagittatum* is 157,114 bp in length, comprising a pair of inverted repeat (IR) regions (25,775 bp) separated by a large single copy (LSC) region (88,507 bp) and a small single copy (SSC) region (17,057 bp). The *E. sagittatum* cp genome contains 133 genes, of which 82 protein-coding genes, 38 tRNA genes, 8 rRNA genes and 5 pseudogenes. Phylogenetic analysis shows that *E*. *sagittatum*is closely clustered with *E*. *lishihchenii*. This genome provides a wealth of information for distinguishing of *Epimedium* species.

*Epimedii Folium*, also known as Yinyanghuo, is an important Chinese traditional medicine. According to the recommendations of the Chinese Pharmacopoeia Commission (2015), four *Epimedium* species were used, including *E. koreanum* Nakai, *E. brevicornu* Maxim., *E. pubescens* Maxim. and *E. sagittatum* (Sieb.et Zucc.) Maxim. The major bioactive components are flavonoid glycosides. (Wang et al. 2007; Ma et al. 2011), *Epimedii Folium* has been verified with activity in nourishing the kidney, reinforcing the Yang, treating osteoporosis, curing cardiovascular diseases, possessing anticancer and anti-aging benefits (Jiang et al. 2015; Wu et al. 2019). *Epimedii sagittatum* is mainly distributed in Eastern and Southern China. The classification and phylogenetic relationship of *Epimedium* has been controversial (Zhang et al. 2016). Previous studies showed that genomic DNA regions are not ideal to identify *Epimedium* species (Guo et al. 2018). However, the chloroplast genome has a conserved sequence ranging approximately 150k bp and providing more variation to discriminate closely related plants (Li et al. 2015). In the present study, we reported the complete chloroplast genome sequence of *E. sagittatum*. The annotated chloroplast genome sequence has been deposited into GenBank with the accession number MN027267.

The total genomic DNA of *E. sagittatum* was extracted from the fresh leaves that were collected in Xinning of Hunan Province, China (N26°33′, E110°48′). The voucher samples (JYZJY01) were deposited at the Herbarium of the Institute of Medicinal Plant (IMPLAD), Beijing, China. Genomic DNA was extracted by using the modified CTAB method (Doyle and Doyle 1987). Total DNA was used for the shotgun library construction. After cluster generation, libraries were sequenced on an Illumina Hiseq 2000 platform and 150 bp paired-end reads were generated. The filtered reads were assembled using the program GetOrganelle v1.5 (Jin et al. 2018) with the reference chloroplast genome of *E. acuminatum* (GenBank: KU522469.1), the chloroplast genome annotation was performed through the online program Dual Organellar Genome Annotator (DOGMA; Wyman et al. 2004) and CPGAVAS (Liu et al. 2012), followed by manual correction.

The complete chloroplast genome of *E. sagittatum* is 157,114 bp in length and contains two inverted repeat (IRa and IRb) regions of 25,775 bp, which were separated by a large single-copy (LSC) region of 88,507 bp and a small single-copy (SSC) region of 17,057 bp. The total GC content of the complete chloroplast genome, LSC, SSC, IR regions is 38.79, 37.38, 32.82, 43.19%, respectively. The complete chloroplast genome harbors 133 genes, including 82 protein-coding genes, 38 tRNA, 8 rRNA genes, and 5 pseudogenes (ψinfA, ψycf1, ψycf15 × 2, ψrpl2). Most of these genes occurred as a single copy. However, trnQ-UUG duplicated in the LSC regions. In addition, four protein-coding genes (rpl23, ndhB, rps7, rps12), seven tRNAs (trnI-CAU, trnL-CAA, trnV-GAC, trnI-GAU, trnA-UGC, trnR-ACG and trnN-GUU), four rRNAs (rrn16, rrn23, rrn4.5, and rrn5) and one pseudogenes (ψycf15) are duplicated in the IR regions. Among these genes, 14 genes (5 tRNA genes and 9 protein-coding genes) contain one intron, and three (ycf3, clpP, and rps12) contain a couple of introns. In these genes, 14 genes (5 tRNA genes and 9 protein-coding genes) contain one intron, and three genes (ycf3, clpP, and rps12) contain a couple of introns. The rps12 gene was trans-spliced, with the 5′ end located in the LSC region and the 3′ end duplicated in the IR region.

To confirm the phylogenetic position of *E. sagittatum*, we downloaded the complete chloroplast genomes of 17 species from the NCBI GenBank database. The nucleotide sequences of the 59 common CDS were extracted from each plastome. The sequences were aligned using MAFFT v7 (Katoh et al. 2017), and RAxML (v8.2.10) (Stamatakis 2014) and were used to construct a maximum likelihood tree, with *Aconitum contortum* and *Coptis chinensis* as the outgroups (Figure 1). Phylogenetic analysis shows that *E*. *sagittatum*is closely clustered with *E*. *lishihchenii*. The published *E. sagittatum* chloroplast genome will provide useful information for phylogenetic and evolutionary studies in Berberidaceae.

**Figure 1.**
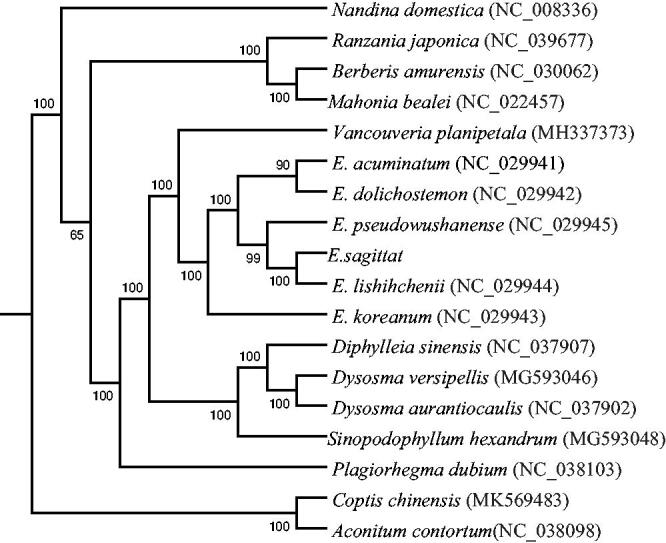
ML phylogenetic tree was constructed based on the 59 common CDS shared between the 18 species.
